# Testing the stress process model in family caregivers of Chinese children and adolescents with depression

**DOI:** 10.3389/fpsyg.2026.1787891

**Published:** 2026-08-12

**Authors:** Xuan Liu, Wei Lin, Hongyan Wu, Lin Lin

**Affiliations:** 1 School of Nursing, Guangdong Medical University, Dongguan, China; 2 Key Laboratory of Lifecycle Care for Chronic Diseases, Guangdong Medical University, Dongguan, China

**Keywords:** anxiety, caregiver burden, caregivers, coping styles, depression, depressive symptoms in children and adolescents, family functioning

## Abstract

**Objective:**

Based on the Stress Process Model (SPM), in this study, we examined the impact of caregiver burden on the mental health of family caregivers of Chinese children and adolescents with depression. We also clarified the mediating effects of coping styles and family functioning within this association.

**Methods:**

We recruited 309 caregivers of children and adolescents with depression from inpatient wards and outpatient clinics at two tertiary hospitals in Guangdong Province, China, between December 2024 and May 2025. We conducted structural equation modeling (SEM) using AMOS 26.0 to examine a serial multiple mediation model.

**Results:**

The total score of caregiver burden was negatively correlated with the scores of the positive coping dimension and the total score of family functioning (*r* = −0.414, −0.391, respectively; *p* < 0.01) and was positively correlated with the scores of dysfunctional coping dimension and the total scores of anxiety and depression (*r* = 0.353, 0.427, 0.433, respectively; *p* < 0.01). We did not observe a significant correlation between caregiver burden with the distraction coping dimension (*r* = 0.041, *p* > 0.05). Caregiver burden indirectly affected caregivers’ anxiety and depression through multiple mediating pathways, including the independent mediating effects of positive coping, dysfunctional coping, and family functioning, as well as the serial mediating effects of positive coping–family functioning and dysfunctional coping–family functioning.

**Conclusion:**

Caregiver burden is directly associated with caregivers’ mental health (anxiety and depression), and may also be indirectly linked via the sequential mediating roles of coping styles and family functioning. This study supports the applicability of the SPM among caregivers of Chinese children and adolescents with depression, and provides empirical support and theoretical foundation for promoting the mental health of caregivers in the Chinese context.

## 1 Introduction

Depression ranks high among the top causes of health-related disabilities on a global scale (Krause et al., 2021), and it significantly adds to the immense workload of caring for people with illness (Muha et al., 2024). Recent epidemiological evidence has indicated a high prevalence rate of depressive disorders in the pediatric and adolescent population. Globally, the prevalence of depressive symptoms ranging from mild to severe in this population has been estimated at approximately 21.3% (Lu et al., 2024). Consistent with these findings, a systematic meta-analysis conducted in China documented that the prevalence of depressive symptoms among children and adolescents was nearly one-fifth, with notable regional variations observed (Rao et al., 2019). Depression in children and adolescents is often accompanied by decreased learning ability, difficulty in getting along with others, and increased risk of self-injury, which make these individuals more dependent on the care and support of their families (Gao et al., 2025; Liu et al., 2024).

In Chinese culture, the care of family members is particularly important for the rehabilitation of adolescents suffering from depression (Wu et al., 2025). Family caregiving constitutes a critical foundation for symptom alleviation and functional recovery in patients; however, the caregiving process is often associated with elevated risks of physical, psychological, and social distress among caregivers (Hu et al., 2024; Phillips et al., 2023). Specifically, while providing care for patients with mental disorders, caregivers often experience long-term negative caregiving experiences. These experiences include negative emotional states, psychological stress, physical exhaustion, decreased quality of life, social isolation, and social discrimination, all of which persist throughout the course of the patient’s disease (Özgönül and Bademli, 2022; Guan et al., 2020; Batool et al., 2024). These multidimensional adverse experiences arising from the caregiving role are commonly conceptualized as “caregiver burden.”

Grad and Sainsbury (1966) first introduced the notion of caregiver burden within the context of providing community-based care for people living with mental health disorders, which they defined as the negative outcomes that family caregivers experience. Mounting empirical evidence has confirmed an association between caregiver burden and caregivers’ mental health. Caregivers with a higher level of caregiving burden are more likely to experience symptoms of anxiety and depression, with the incidence of psychological distress being significantly higher than that experienced by non-caregivers (Soh et al., 2025; Lane et al., 2022). Caregiver burden, however, does not influence mental health outcomes in a simple or direct manner; instead, its effects may be jointly shaped by individual- and family-level resources. Therefore, exploring the potential pathways between caregiving burden and mental health holds significance.

Coping style represents a critical individual-level resource influencing caregivers’ mental health. Coping is defined as the behaviors or cognitive strategies that an individual adopts to alleviate stressors in an adverse environment (Riera-López de Aguileta et al., 2020). When taking care of others, the coping style of caregivers is considered to be the key factor affecting their subjective burden and psychological state. Empirical studies have indicated that caregivers who are exposed to comparable levels of caregiver burden may exhibit markedly different mental health outcomes depending on the coping strategies they employ (Zhang et al., 2024). Specifically, positive coping strategies such as problem-solving and seeking support can positively influence positive affect; in contrast, negative coping strategies such as escape and avoidance can positively influence negative affect (Ren et al., 2022).

Family functioning is widely regarded as a key family-level factor influencing caregivers’ experiences of caregiver burden. Grounded in family systems theory, family functioning reflects the quality of family operations in domains such as communication, role allocation, emotional support, and problem solving (Desquenne Godfrey et al., 2024; Croshie-Burnett and Klein, 2009). Empirical evidence has indicated that well-functioning families are better able to foster cohesion and adaptability, thus enabling caregivers to receive greater emotional support and positive feedback during the caregiving process. This supportive family environment may alleviate caregiver burden and mitigate other negative emotional outcomes, such as depression and anxiety (Leeman et al., 2016; Li et al., 2022; Wen et al., 2025).

Increasing evidence has shown that a close relationship exists among caregiver burden, coping styles, and the mental health of caregivers. For example, among parents with asthmatic children, the burden of caregivers has a negative correlation with positive coping styles and a positive correlation with negative coping styles (Zhao et al., 2020). Further, additional research has suggested that as caregiver burden increases, caregivers are more prone to experiencing anxiety, and depressive symptoms, whereas the adoption of positive coping strategies may buffer these adverse emotional outcomes (García-Alberca et al., 2012).

The Stress Process Model (SPM) has emerged as a broadly adopted theoretical framework in cross-cultural caregiver research because of its ability to integrate multilevel factors, including those at the individual, familial, and societal levels (Yu et al., 2020). This model includes four components: background factors, stressors (including primary stressors and secondary stressors), mediating factors, and health outcomes. According to the SPM, primary stressors can exert a direct impact on caregivers’ health outcomes or have an indirect effect by triggering secondary stressors. Meanwhile, mediating factors may play a mediating role between primary stressors and secondary stressors and between primary stressors and health outcomes (Pearlin et al., 1990). The SPM has been applied to caregivers of patients with various medical issues, including stroke (Pendergrass et al., 2017), schizophrenia (Yu et al., 2020), childhood asthma (Fu et al., 2025), and childhood diabetes (Alruwaili, 2025). Nevertheless, studies focusing on the caregivers of youth suffering from depression remain scarce.

To sum up, caregiver burden (a primary stressor) is associated with poorer family functioning (a secondary stressor) and higher levels of anxiety and depressive symptoms (health outcomes) among caregivers (Unsar et al., 2021). The coping styles adopted by caregivers (mediating factors), directly shape their perceived caregiver burden and are also differentially associated with their mental health outcomes (Zhang et al., 2024). Within the SPM framework, individual-level coping responses are theorized to operate proximally in response to stressors and subsequently shape broader systemic outcomes such as family functioning (Pearlin et al., 1990). Evidence also suggests that dysfunctional or avoidant coping behaviors may progressively undermine family communication and cohesion, thereby impairing overall family functioning (Shao et al., 2020), supporting the ordering of coping styles prior to family functioning in the present model. Although these findings are important, less is known about how these variables function together within a unified framework. This limited understanding may constrain the development of targeted interventions. Moreover, the SPM has not yet been validated among caregivers of children and adolescents with depression in China. Given the unique challenges faced by this population, understanding the interplay among caregiver burden, coping styles, family functioning, and anxiety/depression is essential for improving their mental health. To bridge these gaps and extend previous research, this study was guided by the SPM (Pearlin et al., 1990) and focused on examining the specific sequence of caregiver burden → coping styles → family functioning → mental health among caregivers of children and adolescents with depression in the Chinese cultural context. We further explored the potential mechanisms underlying the association between caregiver burden and mental health and provided empirical evidence for developing family intervention strategies.

We proposed the following hypotheses:

*H1*: Caregiver burden is associated with caregivers’ mental health (anxiety and depression) among children and adolescents with depression.

*H2*: Coping styles play a simple mediating role in the association between caregiver burden and caregivers’ mental health (anxiety and depression).

*H3*: Family functioning plays a simple mediating role in the association between caregiver burden and caregivers’ mental health (anxiety and depression).

*H4*: Coping styles and family functioning play a chain-mediating role in the association between caregiver burden and mental health (anxiety and depression) of caregivers.

## 2 Methods

### 2.1 Participants

Data were collected from December 2024 to May 2025, and we adopted convenience sampling to recruit caregivers of children and adolescents with depression from inpatient wards and outpatient clinics at two tertiary hospitals in Zhanjiang and Dongguan, Guangdong Province, China. Patient selection criteria included the following: (1) age 19 years or younger, and (2) meeting the diagnostic criteria for depressive disorder according to the International Classification of Diseases, 11th Revision (ICD-11). The eligibility criteria for inclusion of caregivers in this study were as follows: (1) age 18 years or older, having provided care for at least 1 month, with only one caregiver selected per patient; (2) being a family member who assumed primary caregiving responsibilities; (3) possessing basic communication skills and adequate reading comprehension ability; (4) securing documented consent for their involvement. Caregivers were excluded if they (1) had a prior diagnosis of cognitive impairment, major physical illness, or psychiatric disorders; (2) were paid caregivers. In all, 340 surveys were disseminated, with 309 being deemed fit for use, resulting in a robust response rate of 90.88%. Consequently, our comprehensive study included a core group with those 309 dedicated caregivers.

### 2.2 Ethics

All participants provided documented consent. The researchers adhered to standardized procedures and predefined inclusion and exclusion criteria. All potentially identifiable personal information was removed to ensure participant anonymity. The study protocol was approved by the Medical Ethics Committee of the First Affiliated Dongguan Hospital of Guangdong Medical University (Approval No. YSYJ202412002).

### 2.3 Measures

#### 2.3.1 Demographic characteristics

The study investigators developed a general information survey instrument for the collection of data on the child’s sex and illness severity, as well as caregivers’ sex, kinship with the patient, educational level, monthly household income, and duration of caregiving. The condition severity of patients was determined based on the physician’s clinical diagnosis, which was abstracted from medical records and categorized into mild, moderate, and severe levels.

#### 2.3.2 Caregiver burden

The Zarit Caregiver Burden Interview (ZBI) was developed by Zarit et al. (1980) based on theories of caregiving burden and is widely used to assess caregivers’ subjective burden. The scale consists of 22 items across two dimensions: personal strain and role strain. Each item is rated on a 5-point Likert scale ranging from 0 (never) to 4 (nearly always), yielding a total score between 0 and 88. Total scores of < 21 indicate little or no burden, scores of 21–39 indicate moderate burden, and scores ≥ 40 indicate severe caregiver burden. The Chinese version of the ZBI has demonstrated good psychometric properties, with a Cronbach’s *α* of 0.87 and satisfactory construct validity (Wang et al., 2006). In the present study, the scale showed excellent internal consistency, with a Cronbach’s *α* of 0.952.

#### 2.3.3 Anxiety

The Self-Rating Anxiety Scale (SAS) was developed by Zung (1971) and is widely used to assess the severity of anxiety symptoms experienced during the past week. The scale consists of 20 items rated on a 4-point Likert scale ranging from 1 to 4, yielding a raw total score between 20 and 80. The SAS standard score is calculated by multiplying the raw score by 1.25 and rounding to the nearest integer, resulting in a standardized score ranging from 25 to 100. Standardized scores of 50–59 indicate mild anxiety, 60–69 indicate moderate anxiety, and scores ≥ 70 indicate severe anxiety, with higher scores reflecting greater anxiety severity. The SAS has demonstrated good reliability and validity, and in the present study, the scale showed excellent internal consistency, with a Cronbach’s *α* coefficient of 0.917.

#### 2.3.4 Depression

The Self-Rating Depression Scale (SDS) was developed by Zung (1965) and is widely used to assess the severity of depressive symptoms experienced during the past week. The scale consists of 20 items rated on a 4-point Likert scale ranging from 1 to 4, yielding a raw total score between 20 and 80. The SDS standardized score is calculated by multiplying the raw score by 1.25 and rounding to the nearest integer, resulting in a standardized score ranging from 25 to 100. According to Chinese normative data, a standardized score ≥ 53 indicates the presence of depressive symptoms, with scores of 53–62 classified as mild depression, 63–72 as moderate depression, and ≥ 73 as severe depression. Higher scores reflect greater severity of depressive symptoms. The SDS has demonstrated good reliability and validity, and in the present study, the scale showed excellent internal consistency, with a Cronbach’s *α* coefficient of 0.920.

#### 2.3.5 Coping styles

The Brief Coping Orientation to Problems Experienced (Brief-COPE) scale is used primarily to evaluate people’s coping strategies when they encounter stress events (Carver et al., 1989). Tang et al. (2021) revised the scale for Chinese caregivers of children with chronic illnesses, resulting in a 28-item version comprising three dimensions: positive coping, distraction coping, and dysfunctional coping. The revised scale demonstrated excellent content validity (S-CVI = 0.97), with a general S-CVI ≥ 0.80 considered satisfactory (Polit et al., 2007). The overall Cronbach’s *α* was 0.83, and subscale α values ranged from 0.57 to 0.89, indicating acceptable reliability. Carver (1997) suggested that alpha scores exceeding 0.50 are acceptable. In this study, the Brief-COPE scale had an overall Cronbach’s α of 0.773, with subscale *α* values of 0.905, 0.664, and 0.833, respectively.

#### 2.3.6 Family functioning

The Family Adaptation, Partnership, Growth, Affection, and Resolve Index (APGAR) scale was developed to evaluate individuals’ perceived level of family functioning (Smilkstein, 1984). The scale has five items, for which higher scores correspond to better family functioning. Total scores of 7–10 indicate that the family is operating well, scores of 4–6 indicate moderate dysfunction, and scores of 0–3 indicate severe serious difficulties. In this study, the APGAR scale had a Cronbach’s α of 0.913.

#### 2.3.7 Control variables

Based on existing research, the following demographic variables were included as control variables: caregiver gender (Ocansey et al., 2021), caregiving duration (Unsar et al., 2021), and condition severity of patients (Wilkinson et al., 2013), as these factors have been identified as correlates of psychological outcomes. Considering the structural equation model complexity, variables with stronger theoretical relevance to the stress process model were prioritized to maintain model parsimony.

### 2.4 Statistical analysis

We entered data using EpiData 3.1 software and parsed the data using SPSS 25.0. We presented data on categorical variables in a frequency and percentage format (n/%) and continuous variables as mean values with standard deviations (mean ± SDs). To evaluate any potential common method variance, we administered Harman’s one-factor test. We conducted structural equation modeling in AMOS 26.0 using maximum likelihood estimation.

## 3 Results

### 3.1 Common method bias test

We separated the factors whose eigenvalues exceed 1. The results showed that the first principal component accounted for only 25.16% of the total variance, which did not reach the critical threshold of 40%. This result indicated that the study did not have any serious common method deviation.

### 3.2 Demographic description

Table 1 presents participant demographics. Caregivers were predominantly female (77.3%), with mothers making up the primary group (69.9%). Many of the caregivers had a junior high school education (35.9%), and 51.5% had a monthly household per capita income in the range of 2,000–5,999 Chinese yuan (CNY). Additionally, 34.0% of caregivers spent 12 or more hours per day caring for the patients. Among the children and adolescents under their care, 202 (65.4%) were female and 107 (34.6%) were male, with severe depression being the predominant severity level (38.9%).

**Table 1:** Characteristics of children and adolescents with depression and their caregivers (*n* = 309).

Variable	*N* (%)
Patient gender	
Female	202 (65.4)
Male	107 (34.6)
Condition severity of patients	
Mild	112 (36.2)
Moderate	77 (24.9)
Severe	120 (38.9)
Caregiver gender	
Female	239 (77.3)
Male	70 (22.7)
Average monthly income per household member (CNY)	
< 2000	48 (15.5)
2000–3,999	79 (25.6)
4,000–5,999	80 (25.9)
6,000–7,999	44 (14.2)
≥ 8,000	58 (18.8)
Kinship with patients	
Father	66 (21.4)
Mother	216 (69.9)
Others	27 (8.7)
Caregivers’ education level	
Primary School and Below	43 (13.9)
Junior High School	111 (35.9)
Senior High School	63 (20.4)
Junior College	61 (19.8)
Bachelor’s Degree and Above	31 (10.0)
Daily care time for patients (hours)	
≤ 5	119 (38.5)
6–11	85 (27.5)
≥ 12	105 (34.0)

### 3.3 Descriptive statistics of variables

Regarding the main study variables, the caregivers’ care burden score was (27.03 ± 18.49), which corresponded to a moderate burden level; the family functioning score was (6.75 ± 2.80), which indicated a generally moderate degree of impairment. The average scores for positive coping, dysfunctional coping, and distraction coping subscales were (2.56 ± 0.65), (2.02 ± 0.61), and (2.23 ± 0.67), respectively, with positive coping being the predominant strategy. The caregivers’ anxiety score was (41.44 ± 12.41). Specifically, 236 cases (76.38%) did not have any anxiety symptoms, 40 cases (12.94%) had mild anxiety symptoms, 26 cases (8.41%) had moderate anxiety symptoms, and 7 cases (2.27%) had severe anxiety symptoms. The caregivers’ depression score was (43.10 ± 13.45), including 229 cases (74.11%) with no depressive symptoms, 52 cases (16.83%) with mild depression symptoms, 23 cases (7.44%) with moderate depression symptoms, and 5 cases (1.62%) with severe depression symptoms. Detailed scores are presented in Table 2.

**Table 2:** Scores of the studied variables.

Variable	Number of items	Range of scores	Total score $\bar{X}\pm S$	Mean item score $\bar{X}\pm S$
Caregiver burden	22	0–88	27.03 ± 18.49	1.23 ± 0.84
Family functioning	5	0–10	6.75 ± 2.80	1.35 ± 0.56
Positive coping	14	14–56	35.86 ± 9.12	2.56 ± 0.65
Dysfunctional coping	10	10–40	20.22 ± 6.06	2.02 ± 0.61
Distraction coping	4	4–16	8.93 ± 2.68	2.23 ± 0.67
Anxiety	20	25–100	41.44 ± 12.41	2.07 ± 0.62
Depression	20	25–100	43.10 ± 13.45	2.16 ± 0.67

### 3.4 Correlations

We conducted Pearson correlation analysis and identified a negative correlation between caregiver burden and both positive coping and family functioning and a positive correlation with dysfunctional coping, anxiety, and depression. Caregiver burden did not have a significant association with distraction coping. Table 3 presents detailed findings.

**Table 3:** Correlations among the studied variables (*n* = 309, r).

Variable	Caregiver burden	Positive coping	Dysfunctional coping	Distraction coping	Family functioning	Anxiety	Depression
Caregiver burden	1	–	–	–	–	–	–
Positive coping	−0.414**	1	-	–	–	–	–
Dysfunctional coping	0.353**	−0.228**	1	–	–	–	–
Distraction coping	0.041	−0.029	0.067	1	–	–	–
Family functioning	−0.391**	0.403**	−0.335**	−0.083	1	–	–
Anxiety	0.427**	−0.531**	0.349**	−0.048	−0.427**	1	–
Depression	0.433**	−0.493**	0.331**	0.048	−0.396**	0.766**	1

**p < 0.01.

### 3.5 Mediation analysis

#### 3.5.1 Coping styles and family functioning as mediators between caregiver burden and anxiety

We tested SEM using AMOS, with caregiver burden as the predictor, anxiety as the outcome, and coping styles and family functioning as the mediators. Parameter estimation occurred through maximum likelihood. This model demonstrated an acceptable fit to the data (*χ*^2^/df = 1.977, IFI = 0.967, GFI = 0.979, CFI = 0.966, TLI = 0.927, RMSEA = 0.056). While most fit indices indicated excellent model fit (CFI, GFI, IFI > 0.950), the TLI was near the commonly accepted threshold (TLI = 0.927). According to widely used criteria (*χ*^2^/df < 3, RMSEA < 0.08, CFI > 0.90, GFI > 0.90, IFI > 0.90, TLI > 0.90) (Łakuta, 2018; West et al., 2012), these values still indicate an acceptable fit. Therefore, the overall model fit can be considered adequate.

**Figure 1:**
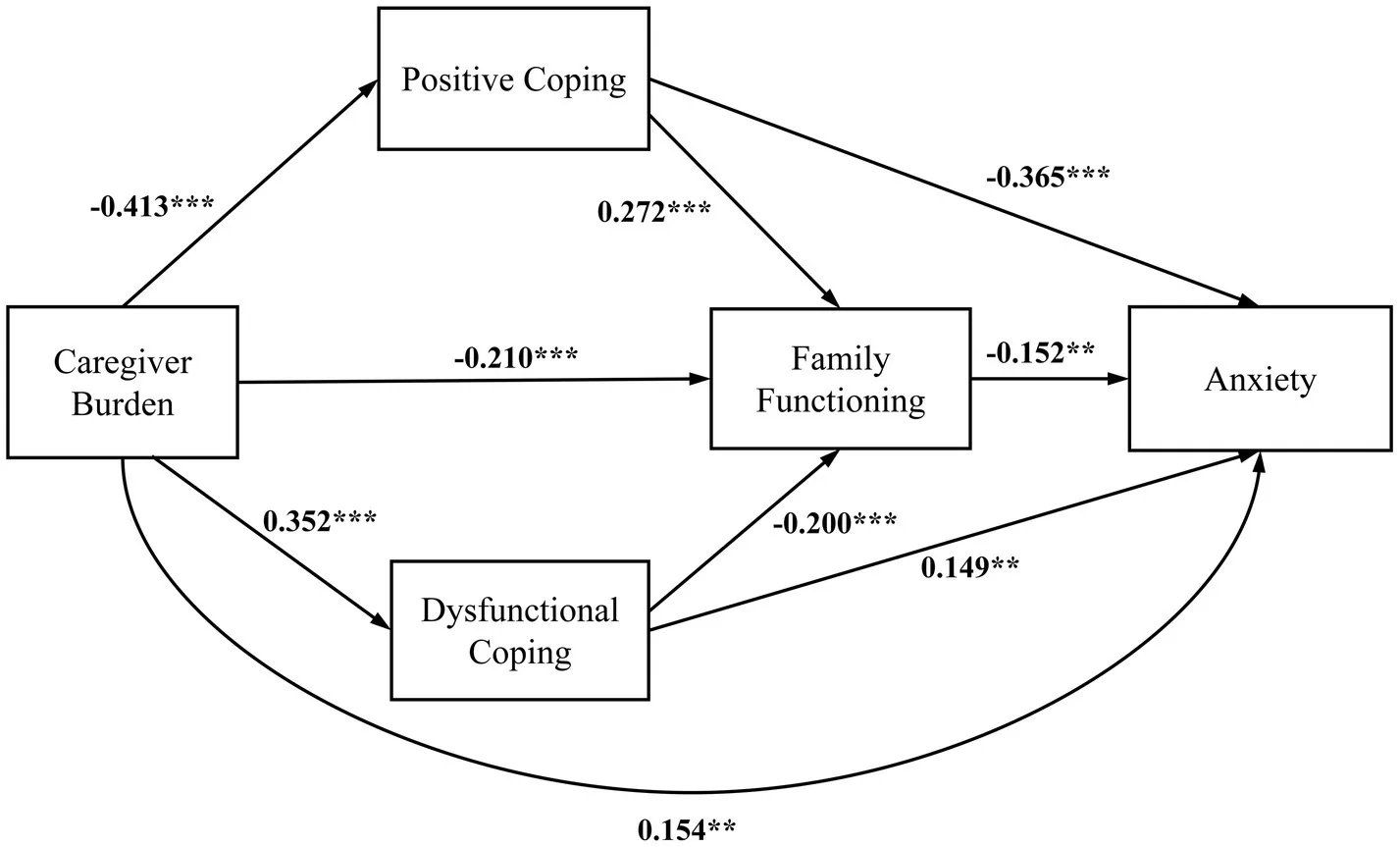
Serial mediation model of coping styles and family functioning in the association between caregiver burden and anxiety (**p < 0.01, ***p < 0.001).

As illustrated in Figure 1, caregiver burden among caregivers of children and adolescents with depression had significant path coefficients with anxiety (*β* = 0.154, *p* < 0.01), and coping styles (*β*_positive coping_ = −0.413, *β*_dysfunctional coping_ = 0.352, *p* < 0.001). Coping styles had significant path coefficients with anxiety (*β*_positive coping_ = −0.365, *β*_dysfunctional coping_ = 0.149, *p* < 0.01). The effect of coping styles as the first mediating variable on the second mediating variable of family functioning was also significant (*β*_positive coping_ = 0.272, *β*_dysfunctional coping_ = −0.200, *p* < 0.001). Family functioning also showed a significant path coefficient with predicted anxiety (*β* = −0.152, *p* < 0.01). All of the path coefficients were statistically significant. The squared multiple correlations (*R*^2^) for key endogenous variables in the model revealed that caregiver burden explained 17.2 and 12.5% of the variance in positive coping and dysfunctional coping, respectively. Together with caregiver burden, positive coping and dysfunctional coping explained 25.1% of the variance in family functioning. Finally, after controlling for caregiver gender, caregiving duration, and condition severity of patients, the full set of predictors explained 38.8% of the variance in anxiety.

We used a bootstrap method with 5,000 replications to calculate the 95% confidence intervals (CIs). The mediation analysis indicated that the direct effect of caregiver burden on anxiety was statistically significant [*β* = 0.111; 95% CI (0.035, 0.187)], the paths through single mediation of positive coping [*β* = 0.107; 95% CI (0.072, 0.150)], and dysfunctional coping [point estimate = 0.038; 95% CI (0.010, 0.069)], and the paths through single mediation of family functioning [*β* = 0.023; 95% CI (0.005, 0.049)], as well as the serial mediating paths through positive coping-family functioning [*β* = 0.012; 95% CI (0.003, 0.024)] and dysfunctional coping-family functioning [*β* = 0.008; 95% CI (0.001, 0.017)], were all statistically significant for the association between caregiver burden and anxiety. Table 4 presents detailed findings.

**Table 4:** Mediation analyses of the association between caregiver burden and anxiety in the SPM (*n* = 309).

Path	Estimate	BootSE	Bootstrap LLCI	Bootstrap ULCI
Caregiver burden → family functioning → anxiety	0.023	0.011	0.005	0.049
Caregiver burden → positive coping → anxiety	0.107	0.020	0.072	0.150
Caregiver burden → dysfunctional coping → anxiety	0.038	0.015	0.010	0.069
Caregiver burden → positive coping → family functioning→anxiety	0.012	0.005	0.003	0.024
Caregiver burden → dysfunctional coping → family functioning → anxiety	0.008	0.004	0.001	0.017
Total indirect effect	0.188	0.028	0.134	0.244
Direct effect	0.111	0.039	0.035	0.187
Total effect	0.299	0.037	0.225	0.372

#### 3.5.2 Coping styles and family functioning as mediators between caregiver burden and depression

We utilized AMOS to build a SEM to test the mediational pathways of caregivers’ coping styles and family functioning in the link between caregiver burden and depression. We defined caregiver burden as the predictor, depression as the outcome, and coping styles and family functioning as mediators. We estimated model parameters using maximum likelihood estimation. The model showed an acceptable fit, with the following indices: *χ*^2^/df = 1.977, IFI = 0.966, GFI = 0.979, CFI = 0.965, TLI = 0.924, and RMSEA = 0.056. While most fit indices indicated excellent model fit (CFI, GFI, IFI > 0.950), the TLI was near the commonly accepted threshold (TLI = 0.924). According to widely used criteria (*χ*^2^/df < 3, RMSEA < 0.08, CFI > 0.90, GFI > 0.90, IFI > 0.90, TLI > 0.90) (Łakuta, 2018; West et al., 2012), these values still indicate an acceptable fit. Therefore, the overall model fit can be considered adequate.

**Figure 2:**
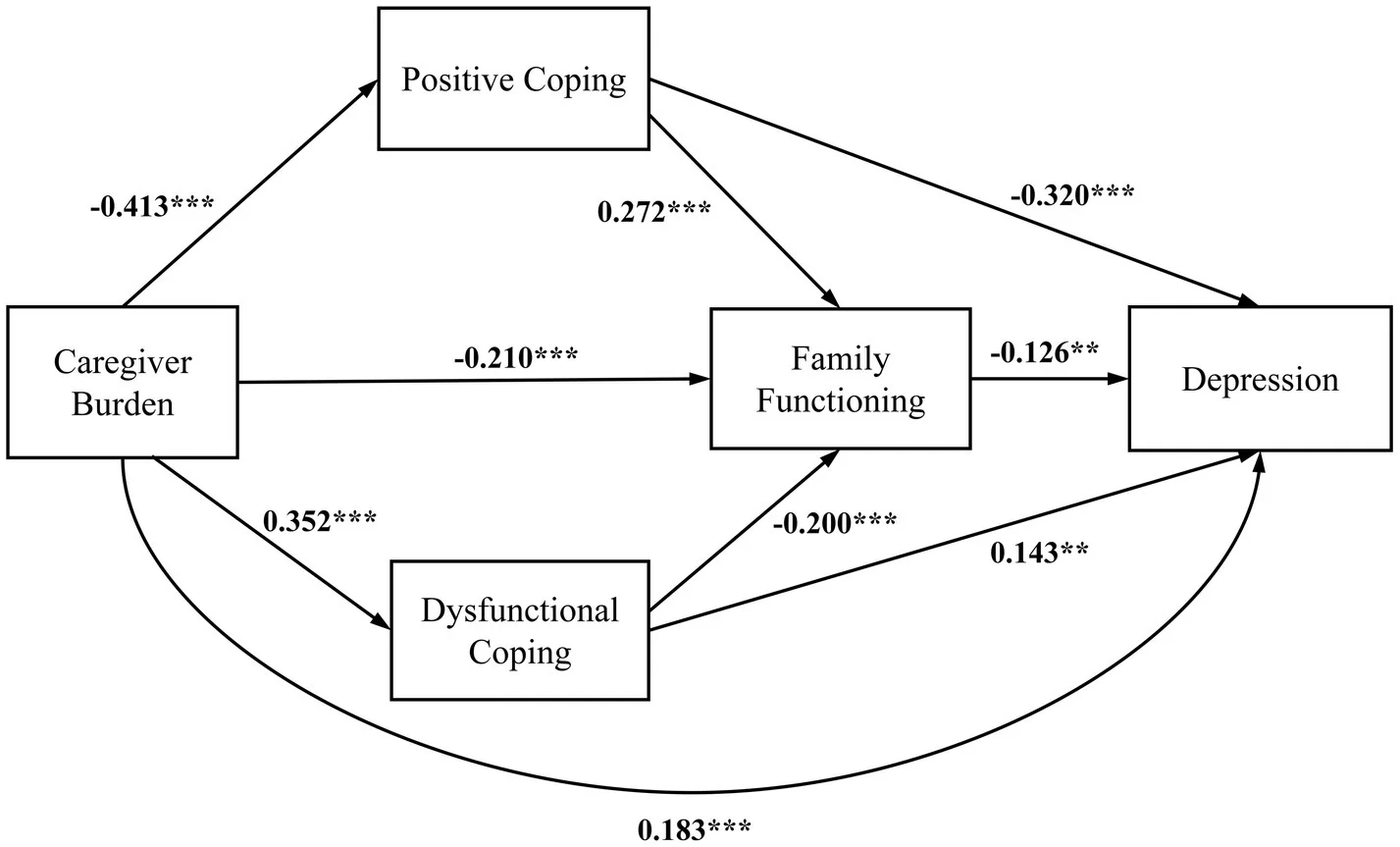
Serial mediation model of coping styles and family functioning in the association between caregiver burden and depression (**p < 0.01, ***p < 0.001).

As illustrated in Figure 2, caregiver burden among caregivers of children and adolescents with depression had significant path coefficients with depression (*β* = 0.183, *p* < 0.001) and coping styles (*β*_positive coping_ = −0.413, *β*_dysfunctional coping_ = 0.352, *p* < 0.001). Coping styles, in turn, had significant path coefficients with depression (*β*_positive coping_ = −0.320, *β*_dysfunctional coping_ = 0.143, *p* < 0.01). The effect of coping styles as the first mediating variable on the second mediating variable of family functioning was also significant (*β*_positive coping_ = 0.272, *β*_dysfunctional coping_ = −0.200, *p* < 0.001). Family functioning also showed a significant path coefficient with predicted depression (*β* = −0.126, *p* < 0.01). All of the path coefficients were statistically significant. The *R*^2^ values for key endogenous variables in the model revealed that caregiver burden explained 17.2 and 12.5% of the variance in positive coping and dysfunctional coping, respectively. Together with caregiver burden, positive coping and dysfunctional coping explained 25.1% of the variance in family functioning. Finally, after controlling for caregiver gender, caregiving duration, and condition severity of patients, the full set of predictors explained 35.8% of the variance in depression.

We conducted bootstrap resampling with 5,000 iterations to estimate the 95% CIs for the mediating effects. The mediation analysis indicated that the direct effect of caregiver burden on depression was statistically significant [*β* = 0.143; 95% CI (0.059, 0.227)], the paths through single mediation of positive coping [*β* = 0.101; 95% CI (0.064, 0.145)], dysfunctional coping [*β* = 0.040; 95% CI (0.011, 0.073)], and the paths through single mediation of family functioning [*β* = 0.021; 95% CI (0.003, 0.043)], as well as the serial mediating paths through positive coping-family functioning [*β* = 0.011; 95% CI (0.002, 0.023)] and dysfunctional coping-family functioning [*β* = 0.007; 95% CI (0.001, 0.016)], were all statistically significant for the association between caregiver burden and depression. Table 5 presents detailed findings.

**Table 5:** Mediation analyses of the association between caregiver burden and depression in the SPM (*n* = 309).

Path	Estimate	BootSE	Bootstrap LLCI	Bootstrap ULCI
Caregiver burden → family functioning → depression	0.021	0.010	0.003	0.043
Caregiver burden → positive coping → depression	0.101	0.020	0.064	0.145
Caregiver burden → dysfunctional coping → depression	0.040	0.016	0.011	0.073
Caregiver burden → positive coping → family functioning → depression	0.011	0.005	0.002	0.023
Caregiver burden → dysfunctional coping → family functioning → depression	0.007	0.004	0.001	0.016
Total indirect effect	0.180	0.028	0.128	0.237
Direct effect	0.143	0.043	0.059	0.227
Total effect	0.323	0.040	0.244	0.402

## 4 Discussion

In this current research, the average total care burden among the caregivers measured (27.03 ± 18.49), with an average item score of (1.23 ± 0.84), which reflected a moderate level of care-related stress. These results aligned with the results reported by Song in a study of caregivers of adolescents with depression (Song, 2024), but they were lower than the level observed by Liu among caregivers of patients with depression (Liu, 2022). Variations in the age of care recipients may account for this observed discrepancy. Children and adolescents with depression are generally younger and tend to have a shorter duration of disease, with a relatively favorable prognoses. In addition, they can often benefit from support provided by multiple systems, including family and school, which collectively may alleviate caregivers’ perceived burden. However, 56.31% of caregivers in this study experienced moderate to severe caregiver burden, suggesting a substantial heterogeneity in burden distribution, with a considerable proportion of families facing pronounced caregiving stress. These findings indicated that in clinical practice, differentiated support strategies need to be adopted. Although general support measures remain crucial, more attention should be paid to those caregivers who bear moderate to severe burdens. Their potential risk factors should be identified and targeted intervention measures should be implemented.

For the present study sample, the mean score on the positive coping dimension was (2.56 ± 0.65), which was higher than that for dysfunctional coping (2.02 ± 0.61) and distraction coping (2.23 ± 0.67). Among the three coping styles, positive coping exhibited the highest level, which was consistent with findings from previous research (Du et al., 2025; Kim and Jang, 2025). This pattern may be related to the predominance of parents in the study sample. As primary caregivers, parents are often socially assigned strong moral responsibilities and positive role expectations. This kind of role identification, coupled with cultural rules, may relate to more frequent use of positive coping strategies. Parents tend to find ways to solve problems, ask for help, and make plans more often.

In this study, the mean total family functioning score among caregivers was (6.75 ± 2.80), which indicated a moderate level of family dysfunction. Notably, 46.28% of caregivers experienced moderate to severe family dysfunction. The total family functioning score in this study was comparable to the score reported by Lee et al. (2023). These findings may reflect suboptimal levels of intimacy, cooperation, and emotional connectedness among family members of patients with depression, with some families also experiencing interpersonal conflicts (Du et al., 2025). These results may also be related to the stress experienced by families caring for a child with depression (Factor et al., 2022). Previous research has shown that parent–child communication plays a pivotal role in maintaining family functioning and that mutual understanding and support among family members can make it easier to cope with the challenges posed by childhood and adolescent depression (Pontes et al., 2023). Therefore, these findings underscore the importance of incorporating family functioning assessments into clinical practice and provide reference for future studies in this field.

In this study, the mean total anxiety score among caregivers was (41.44 ± 12.41), and the mean total depression score was (43.10 ± 13.45), which were higher than those reported by Li (2023). In addition, based on test results with values above the minimum critical threshold indicating positivity, the positive rates for anxiety and depression were 23.62 and 25.89%, respectively, indicating that some caregivers are still experiencing negative emotions. Parents are the primary caregivers of children and adolescents with depression, and their children’s illness creates significant psychological pressure. Moreover, the prolonged treatment course of depression, its propensity for relapse, and its impact on patients’ academic and social functioning are frequently observed alongside substantial parental stress and variations in emotional well-being. In addition, caregivers may face practical challenges during the caregiving process. For example, some fathers (mothers) may be forced to resign from employment to provide full-time care. Assuming sole responsibility for caregiving can further compromise caregivers’ emotional well-being (Karimi Moghaddam et al., 2023). Given these observed associations, it may be valuable for healthcare professionals to consider not only average levels of anxiety and depression, but also the identification of caregivers at elevated risk, as the present findings suggest that timely psychological support and social resources could be potentially beneficial for this population, pending confirmation through intervention-based research.

The present study further explored the association between caregiver burden and caregivers’ mental health. Caregiver burden is associated with caregivers’ mental health, which confirms Hypothesis 1. A meta-analysis has also confirmed a positive correlation between caregiver burden and anxiety across different caregiver populations (del-Pino-Casado et al., 2021). This may be attributed to the fact that caregivers are required to provide comprehensive support for children with illnesses over the long term, including daily care and follow-up medical visits. The sustained investment of time and energy is frequently linked to a greater sense of burden and elevated psychological stress. Meanwhile, higher caregiver burden is often associated with increased role conflicts, which may make it more challenging for caregivers to balance caregiving responsibilities with personal life and work demands. Combined with factors such as financial strain and social isolation, these challenges tend to be linked with elevated anxiety symptoms. Abdelhalim et al. (2024) conducted a study among 141 family caregivers of individuals with dementia and demonstrated a direct association between caregivers’ subjective caregiver burden and depressive symptoms. This may be associated with the fact that prolonged caregiving tasks occupy a substantial amount of caregivers’ time and energy, which is related to reduced participation in social and leisure activities, and it is further linked to a certain degree of social isolation and feelings of loneliness. Existing research has confirmed that loneliness is a significant predictor of depression (Hymas et al., 2024). These findings highlight the importance of paying attention to caregiver burden. Future research should further explore approaches to alleviating caregiver burden, as they may be relevant to improving mental health.

Beyond the direct association between caregiver burden and mental health, the present study further explored the potential mediating role of coping styles within this relationship. The significant independent mediation by coping styles (H2) aligns with theoretical and empirical links between caregiver burden, coping styles, and anxiety/depression (Lu et al., 2017; Ay et al., 2017; Pearlin et al., 1990). Both positive coping and dysfunctional coping may serve as potential mediators in the association between caregiver burden and anxiety/depression. Coping refers to the process by which individuals manage stress, and coping styles are closely associated with psychological health outcomes. Previous research has shown that under high-stress conditions, individuals who lack effective coping strategies may face a risk of psychological impairment as high as 43.3%, which is approximately twice the corresponding rate observed among the general population (Andrews et al., 1978). On the one hand, the heavy burden of caregiving is linked to depletion of caregivers’ psychological resources, which may in turn be linked to a greater likelihood of adopting dysfunctional coping strategies, such as denial, avoidance, behavioral withdrawal, self-blame, or substance abuse. While these methods may provide temporary relief from stress, they often fail to address the underlying problems. There is a tendency for individuals who rely more on such strategies to also report higher levels of psychological depletion and depressive symptoms. On the other hand, caregiver burden was also associated with the use of positive coping mechanisms, such as acceptance of reality, seeking support, positive reframing, and relying on faith. Use of these strategies tends to coincide with reduced inner conflict, more positive perceptions, and improved emotion regulation among caregivers.

From a theoretical perspective, within the SPM, not all coping strategies play an equivalent role in the association between caregiver burden and psychological outcomes. In this study, distraction coping showed no significant correlation with caregiving burden, family functioning, or depression. Distraction coping, which encompasses self-distraction and humor, may be particularly less adaptive in Chinese caregiver populations for several reasons. First, self-distraction is often associated with behavioral withdrawal and can be considered a form of avoidant coping, which is generally not effective for problem resolution (Ren et al., 2022). Second, humor is less commonly employed as a coping strategy in Chinese cultural contexts compared with Western populations, and different forms of humor may exert divergent effects on psychological well-being (Tang et al., 2021; Chen and Martin, 2007), potentially resulting in null effects when these subtypes are aggregated into a single composite score. Consequently, distraction coping as a whole may lack the adaptive capacity to mediate the relationship between caregiving burden and depression in this population. The present findings thus refine our understanding of the differential roles of coping strategies within the stress process model, and they suggest that future research should examine self-distraction and humor as distinct constructs, with explicit attention to cultural context when applying coping frameworks to Chinese caregiver samples.

Family functioning, as a significant independent mediator, supported Hypothesis 3. Consistent with previous studies, our findings indicated that well-functioning families were generally associated with lower levels of caregiving burden (Yu et al., 2021), and family functioning was also negatively associated with anxiety and depression (White Makinde et al., 2025; Shao et al., 2020). The family represents the most fundamental unit of human life, and its normal functioning is particularly important for physical and mental health. During the caregiving process, family functioning may be at lower levels under caregiving stress, and caregivers may not receive sufficient psychological or practical support, which may be related to higher levels of anxiety and depression among caregivers. Therefore, healthcare professionals should consider family functioning as a key assessment indicator to identify potentially high-risk families and to explore appropriate support strategies.

Furthermore, we examined the serial mediating pathway involving coping styles and family functioning. Statistical support was found for the hypothesized chain mediation pathway (caregiver burden → coping styles → family functioning → anxiety/depression), providing support for Hypothesis 4. These findings are consistent with the theoretical predictions of the SPM, suggesting that coping styles and family functioning may sequentially link caregiver burden to mental health outcomes. Previous research has identified a strong association between coping styles and family functioning (Clari et al., 2022). Family functioning is an important factor associated with caregivers’ coping strategies. Additionally, coping strategies are key aspects of the caregiving process, which can affect the operation of the family. When caregivers are particularly stressed, they may use negative strategies to deal with it, such as avoidance or self-criticism. These methods may be less effective and are often observed alongside poorer emotional connections, role ambiguity within the family, weakened support networks, and reduced family functioning. Moreover, dysfunctional coping was associated with higher levels of perceived caregiver burden and greater feelings of fatigue, and it may be related to increased negative emotions. When caregivers are able to adopt proactive coping strategies in response to stress, such as seeking emotional support, actively participating in problem-solving, and redefining the caregiving experience, the family function is more likely to remain stable, and caregivers’ mental health is more likely to be protected. These results showed that it is clinically significant to evaluate the burden of care, enhance positive coping resources, and implement targeted intervention measures aimed to improve family function.

Given the cross-sectional design of this study, we can only identify correlational and mediational pathways, but we cannot establish definitive causal relationships among caregiver burden, coping styles, family functioning, and anxiety/depression. Moreover, regional socioeconomic and healthcare contexts may shape the stress process among caregivers. Given that participants in this study were recruited from Guangdong Province, an economically developed coastal region of China, the availability of medical resources, financial conditions, and social support may influence caregivers’ coping strategies and family functioning. Therefore, the strength and structure of the observed mediation pathways may differ in regions with fewer healthcare and social resources.

## 5 Strengths and limitations

This study has several limitations. First, the cross-sectional design precludes any causal inferences among the variables. Future research should adopt longitudinal or interventional designs to better establish causal relationships. Second, the reliance on self-reported data introduces potential reporting bias, as responses may be influenced by personal biases or inaccurate recall. In addition, common method bias was assessed using only Harman’s one-factor test, which is generally considered insufficient; future studies should adopt more rigorous approaches, such as the CFA marker technique, to better address this issue. Third, participants were recruited through convenience sampling and were restricted to caregivers of children and adolescents with depression from two tertiary hospitals in Guangdong Province, China. As a coastal province, Guangdong may differ from inland regions in medical resource availability, economic development, and local policy support. These factors may influence caregivers’ anxiety and depression levels, thereby limiting the generalizability of the findings. Future research should expand the sampling scope to include diverse geographic regions and medical institutions at different levels in China to improve representativeness and external validity. Fourth, although previous studies suggest that family functioning may reciprocally influence coping styles or that the two may have bidirectional relationships, this study—guided by the SPM framework—only examined a specific sequential path (coping styles → family functioning). Future studies are needed to explore other possible sequential relationships among these variables.

Despite these limitations, this study has several strengths. First, among Chinese caregivers of children and adolescents with depression, it provides a new theoretical perspective by clarifying how caregiver burden, coping styles, family functioning, and mental health operate as an integrated system. Second, the serial mediation model reveals a chained pathway—caregiver burden → coping styles → family functioning → anxiety/depression—thereby enhancing the explanatory power of the SPM. Third, although similar serial mediation models have been tested in some caregiver populations (Saffari et al., 2018; Wen et al., 2022), this specific chained mechanism has rarely been examined among caregivers of children and adolescents with depression. This research thus provides a new perspective from both theoretical and practical aspects, which is helpful when formulating more effective treatment plans for adolescent patients with depression and their caregivers.

## 6 Conclusion

In this study, we validated the SPM among the caregivers of Chinese children and adolescents with depression, systematically explored the association between caregiver burden and anxiety or depression, and revealed the mediating roles of coping styles and family function in these associations. Future research should explore more mediating factors to comprehensively understand the associations between caregiver burden and mental health. Moreover, healthcare providers should pay attention to caregiver burden, cultivate positive coping strategies, implement effective measures to promote family functions, and thereby enhance the mental health of caregivers.

## Data Availability

The raw data supporting the conclusions of this article will be made available by the authors, without undue reservation.
